# Application of one health approach in training at Makerere University: experiences from the one health workforce project in Uganda

**DOI:** 10.1186/s42522-020-00030-7

**Published:** 2020-11-30

**Authors:** Edwinah Atusingwize, Rawlance Ndejjo, Gloria Tumukunde, Esther Buregyeya, Peninah Nsamba, Doreen Tuhebwe, Charles Drago Kato, Irene Naigaga, David Musoke, John David Kabasa, William Bazeyo

**Affiliations:** 1 Makerere University School of Public Health, Kampala, Uganda; 2 Makerere University College of Veterinary Medicine, Animal Resources and Biosecurity, Kampala, Uganda; 3 One Health Central and Eastern Africa (OHCEA), Kampala, Uganda

**Keywords:** One health, Multidisciplinary, Training, Workforce development, Professional development, Uganda

## Abstract

The interconnections of humans, domestic animals, wildlife and the environment have increasingly become complex, requiring innovative and collaborative approaches (One Health approach) for addressing global health challenges. One Health is a multidisciplinary and multi-sectoral collaborative approach to human, animal, plant and environmental health. The role of academia in training professionals oriented in One Health is critical in building a global workforce capable of enhancing synergies of various sectors in improving health.

Makerere University, Uganda has implemented pre-service capacity building initiatives aimed to foster One Health competencies among students who are future practitioners. In addition to incorporating the One Health concept in didactic curricula, Student One Health Innovation Clubs, undergraduate field placements in 11 demonstration sites, graduate fellowships, small grants to support research and innovations, and cross-college collaborative training approaches have greatly aided the assimilation of One Health into the fabric of university offerings. Partnerships with government ministries, private sector and international agencies were initiated to benefit the students, as well as chart a path for experiential learning and in-service offerings in the future.

One major challenge, however, has been the tendency to focus on infectious diseases, especially zoonoses, with less consideration of other health issues. The opportunity for improvement, nonetheless, lies in the increasing emerging and re-emerging health concerns including epidemics, environmental pollution and related challenges which justify the need for countries and institutions to focus on building and strengthening multidisciplinary health systems.

## Background

The complex interconnection of humans, animals (domestic and wild) and their respective social and ecological environment is evident in the current global health challenges which warrant critical attention to be focused on integrated approaches to health protection and promotion [1, 2]. As the human population continues to increase across the world, considering the interconnectedness of people, animals and the environment becomes more important [3, 4], especially in the control of emerging and re-emerging diseases such as zoonoses [5]. In addition, there are many other emerging public health concerns such as trauma, injuries and disabilities and non-communicable diseases [5–8] that all require attention.

Addressing such health issues from a single sector without considerations of the complexity of the entire system (humans, animals, plants and the environment) can be slower and costly [9, 10]. Innovative approaches, including working in collaboration across sectors, are therefore important in addressing the complex challenges that the world is facing today. One Health is a collaborative, multisectoral, and transdisciplinary approach—working at the local, regional, national, and global levels—with the goal of achieving optimal health outcomes, recognizing the interconnection between people, animals, plants, and their shared environment [11, 12]. Important to note is that the One Health approach extends to research, training and service delivery, focused not only on diseases, but also on health at individual, population and ecosystem levels [13–15]. Despite the need for broad integration, it is still common to find efforts towards disease control being implemented from a single sector perspective.

As the One Health approach is gaining global embrace, the role of academia in training future professionals is critical in building a global workforce capable of enhancing synergies across various sectors in improving health. However, research and teaching in institutions of higher learning, including universities, has for a long time been conducted from a single-discipline approach, producing graduates who are professionals in their field with less knowledge on the importance of other sectors and disciplines [16]. Indeed, while the One Health approach is not new, there is evidence of challenges, including its operationalization [17, 18]. However, academic institutions remain important avenues for promoting One Health by creating an enabling environment for training, research and practice [19].

With the purpose of building a multidisciplinary health workforce, the One Health Central and Eastern Africa (OHCEA), an international university network (12 schools of public health, ten veterinary higher education institutions, one institute of environmental sciences, and one pathobiology institute located in 16 universities in 8 countries in the Eastern, Central and Western African regions) [20], through its One Health Workforce (OHW) project, promoted the One Health approach primarily through university training. At Makerere University, initiatives supported by the OHW project aimed to enable students to achieve critical One Health competencies and to implement the approach in their future careers. Our paper describes these One Health initiatives.

### One health initiatives at Makerere University

With the aim of producing One Health graduates with transformative knowledge that can contribute to preventing, detecting and responding to infectious disease outbreaks, OHCEA implemented initiatives that are building a competent One Health workforce, targeting the pre-service level. In addition to incorporating One Health concepts in the didactic curricula, other pre-service capacity building initiatives involved all colleges and included: the Makerere University Students’ One Health Innovation Club (MAKSOHIC); the development of a One Health Institute (OHI) (One Health theoretical principles, field placements, and fellowships); small grants, research and innovations; and collaborative teaching as described in detail below.

#### Makerere University students one health innovation Club (MAKSOHIC)

Using a platform in which students from different disciplines convened for innovative intellectual debate and engagement around identified One Health challenges, participants developed skills and competences in One Health leadership, collaboration and teamwork, community engagement, research, innovation and scientific communication. Open to scholars at all Makerere University colleges, over 560 students from academic disciplines, such as veterinary medicine, environmental health, engineering, social sciences, agriculture and nursing have been involved (40% from College of Veterinary Medicine, Animal Resources and Bio-Security (CoVAB), 25% from College of Health Sciences (CHS), 10% from College of Humanities and Social Sciences (CHUSS), 10% from College of Engineering Design, Art and Technology (CEDAT), and the rest from other colleges).

The club led by seven elected student members, has a constitution which was developed for and by the club members. It is mentored by University faculty patrons and builds students’ skills during hands-on community engagement activities, such as outbreak investigation and rapid response, risk communication and public sensitization on priority zoonotic diseases (e.g., rabies, Ebola, Rift Valley Fever, plague) which are part of the Global Health Security Agenda (GHSA) [21]. Students’ participation in national response to disease outbreaks in Ugandan communities are summarized in Table 1.

**Table 1:** Summary of outbreaks in Uganda in which Students One Health Innovation Club members have been involved

Outbreak	Period	Region (District)	Activities
Rift Valley Fever	February 2016	Western (Kabale)	Identification of missed cases including contact tracing; Outbreak characterization; Case data collection and analysis; Risk factor assessment through interviewing suspected cases; Line listing of suspected cases; Report writing; Post outbreak evaluation on Interventions of Rift Valley Fever
Yellow Fever	April 2016	Central (Masaka)	Identification of missed cases including contact tracing; Outbreak characterization; Case data collection and analysis; Risk factor assessment through interviewing suspected cases; Line listing of suspected yellow fever cases; Report writing
Highly Pathogenic Avian Influenza (HPAI)	February 2017	Central (Shores of Lake Victoria: Masaka, Mukono, Nakasongola, Wakiso, Kalagala)	Social mobilization and sensitization; Stakeholder coordination meetings; Risk and impact assessment of HPAI; Sample collection; Infection control training; Report writing
Anthrax High Alert	July 2017	Northern (Arua)	Epidemiological study to assess factors associated with anthrax outbreak, including community knowledge, attitudes and practices regarding the outbreak; Identification of active human cases and animal deaths due to anthrax; Report writing
Crimean Congo hemorrhagic fever (CCHF)	September 2017	Central (Kiboga and Nakaseke)	Risk assessment of CCHF in livestock; Post evaluation of case management in the hospitals that handled the CCHF cases; Report writing
Marburg	October–November 2017	Eastern ***(***Kween and Kapchorwa)	Designing active case search tool and systematic case finding including entry and management of the active case search logs and contacts listed; Daily situational reports; Cross-border surveillance; Community sensitization using film vans; Receiving/responding to alerts of suspected cases; Record review (passive surveillance) in health centers; Assessment of water sanitation & hygiene, infection control and prevention (IPC) standards in health facilities; Engagement of traditional healers as a key social and belief structure of the affected communities; Orientation in basic IPC drills for field surveillance teams

**Fig. 1:**
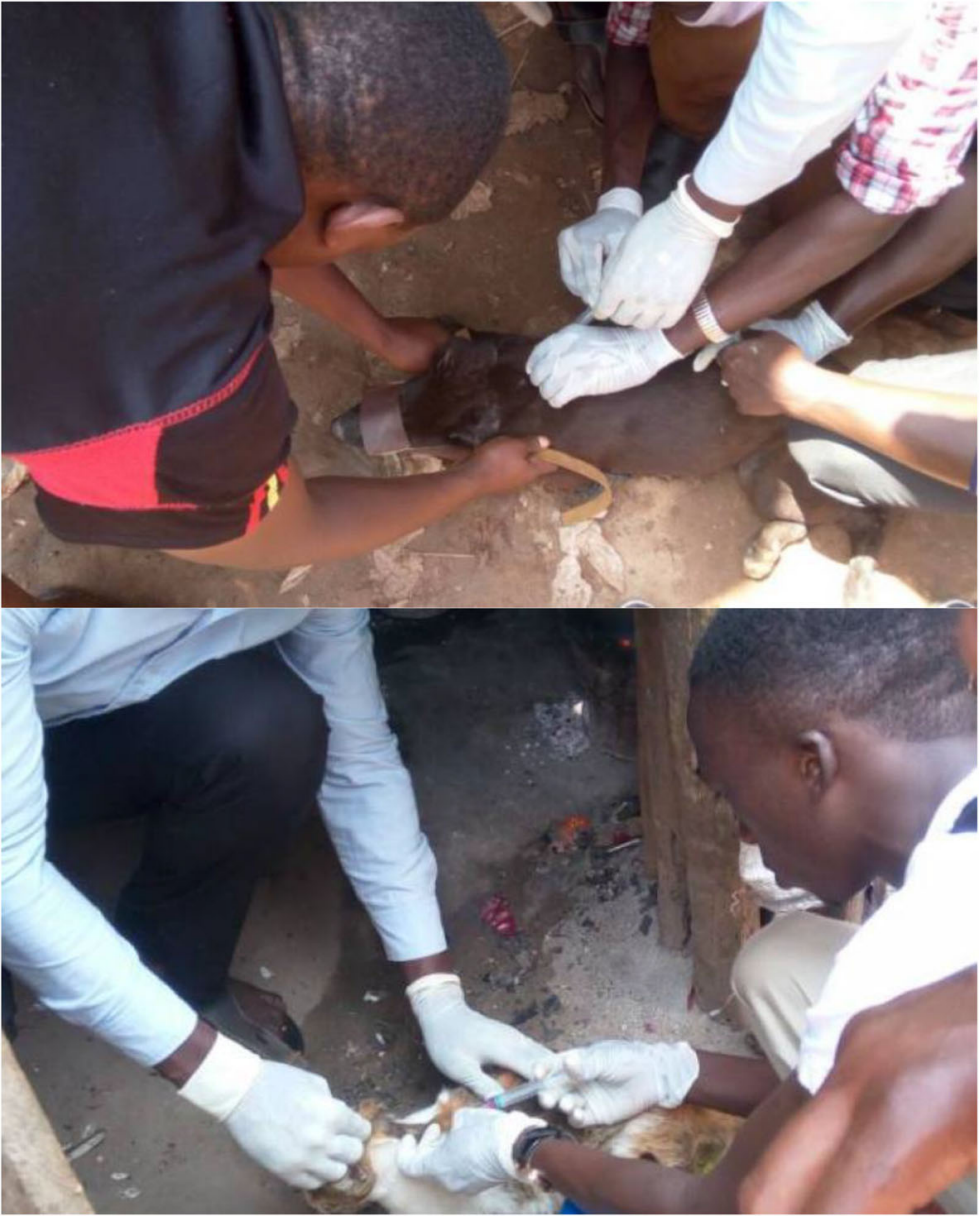
Students One Health Innovation Club members during a vaccination campaign in some areas of Kampala city, 2017 that won a global competition in One Health

Other interventions in communities included mass vaccination of dogs and cats against rabies (Fig. 1), rabies prevention awareness for school going children in Kampala city, deworming of domestic animals, and sensitization on zoonotic diseases among meat handlers in abattoirs. Students’ engagement in these activities was important for building their capacity in leadership, collaborative approaches, awareness campaigns and community engagement, including mobilization and sensitization. During such community engagements, students joined other partners whose contribution to their training activities has been critical. Key partners included the National One Health Platform [a collaboration between the Ministry of Health, Ministry of Agriculture Animal Industry and Fisheries, Uganda Wildlife Authority and Ministry of Water and Environment and other agencies/entities including Food and Agriculture Organization of the United Nations and research institutes, such as the Uganda Virus Research Institute.

#### One health institute

The development of a One Health Institute improved multidisciplinary training on management of infectious diseases for undergraduate and graduate students. In the Institute, facilitators from different specialties and disciplines engaged students through didactic instruction on theoretical principles of One Health, followed by experiential learning through field placements and fellowships.

- One Health theoretical principles

One Health theoretical principles were explored through modules designed to provide the multidisciplinary participants with transformative knowledge, fostering abilities to prevent and mitigate risks at the animal-human-ecosystems interface. The modules were offered to both undergraduates and postgraduates, and participants were competitively selected from different colleges and disciplines within the University, including public health, social sciences, agriculture, veterinary medicine, zoology, biomedical lab technology and economics. The seven modules offered were: (i) Leadership in Infectious Disease Management; (ii) Gender and Risk Management; (iii) Health Policy Analysis; (iv) Bio-risk Management; (v) Disease outbreak Investigation and Emergency Response; (vi) Antimicrobial Resistance and (vii) Community Engagement. Participants were also challenged to innovatively think about problems in terms of entrepreneurship, a socio-innovation concept that they later applied when addressing community challenges during field placements. The multidisciplinary delivery model involved group discussions, role playing, case studies and simulations.

b) Undergraduate field attachments

Field attachments aimed to equip students with hands-on experiences, while giving them an opportunity to apply taught course concepts. In the attachments, groups of students from different disciplines were assigned to one of the eleven One Health field demonstration sites in Uganda for a period of 2–4 weeks. Students conducted problem prioritization and stakeholder identification and engagement, and developed daily action plans through student-led discussions. They were required to carry out projects aimed at addressing a community problem using limited resources, during which all students worked together to suggest appropriate interventions grounded from their disciplines, as well as implementation of a multidisciplinary solution. Highlights of projects from these placements include: using locally available resources to develop repellents to expel bats from infested households and institutions; water treatment systems for use at the household level; and novel hand washing facilities. In another example, a food market in Hima Township of Kasese District had a problem of accumulated charcoal dust residue. In response, a team of students led a community session on how to recycle this waste (charcoal dust) into briquettes, which also served as an additional solution to rising energy demands in the community. Following the attachments, the teams successfully wrote reports and disseminated findings to the district officials, local communities and other key stakeholders to ensure sustainability and ownership of interventions.

c) Graduate fellowships

The One Health Institute also offered multidisciplinary fellowships for graduate students using a mentorship-training model. Following discipline-specific training in their respective programmes and the One Health theoretical principles, graduate students were placed at selected partner organizations and assigned two mentors (academic and field) to support and guide them to acquire One Health competencies. These competencies included problem-solving, multisectoral communication, community engagement, proposal writing, scientific writing and publication. Some of the practical tasks the fellows completed offered important learning opportunities in: submission of financial requisitions, budgeting, acquisition of supplies, collection of samples, submission to laboratories, teambuilding, moderating of discussions, and scientific and financial reporting.

As a primary training activity, fellows conducted a situation analysis at the organization of placement to identify a problem that could be solved using the One Health approach and their newly-acquired competencies, and proposed an intervention to aid the community. Specific technical competencies acquired included Monitoring and Evaluation of OHCEA Field Attachment 2017 and Amref-Health Africa; data analysis at the Uganda National Laboratory Systems involving disease investigation; risk analysis and mapping disease hotspots and zoonoses with the Food and Agriculture Organization; Human Centered-Design-Thinking with the Resilient African Network; drafting training manuals on antimicrobial resistance and assessment of bio-risk management with the Infectious Diseases Institute. In addition to their experiential training and participation in field activities, each One Health fellow was asked to offer routine services of the organization in which they had been placed under close supervision and guidance of the organizational field supervisor. Some of these placements then led to long-term work opportunities for participants by linking them or absorbing them into organizations involved in their fellowships. A summary of the graduate fellow completion statistics is shown below (Table 2).

**Table 2:** Summary of graduate fellows

OHI Graduate Fellowship				
Intake Year	No. awarded	Completed (%)	Male	Female
**2017**	8	8 (100%)	4 (50%)	4 (50%)
**2018**	10	10 (100%)	5 (50%)	5 (50%)
**2019**	6	4 (67%)	4 (67%)	2 (33%)
**Total**	**24**	**92 (78.4%)**	**13 (54%)**	**11 (46%)**

#### Small grants, research, and innovations

**Fig. 2:**
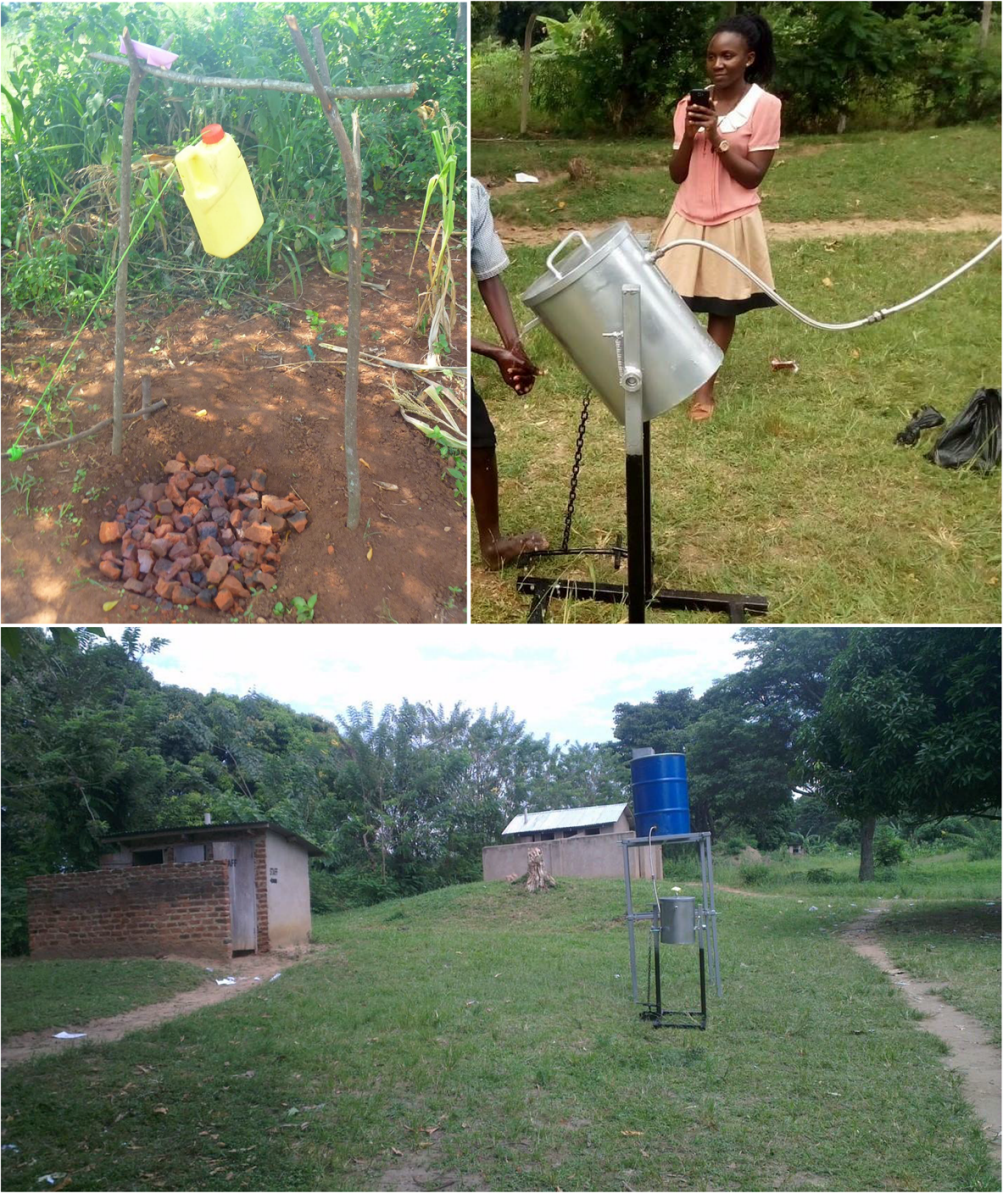
Traditional *tippytap (left),* and an improved *tippytap* developed by students *(right)*, 2016

**Fig. 3:**
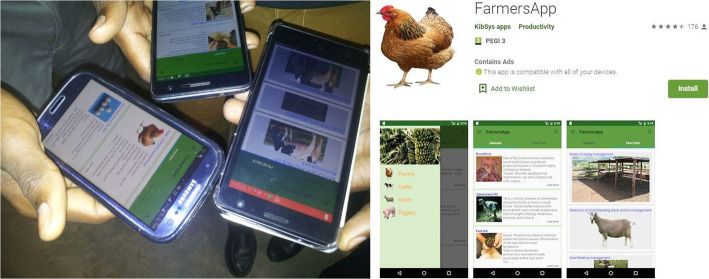
Student demonstrating the *Farmers App (left)* and the different views of the app *(right)*

The OHW project offered grants for undergraduate and graduate students to conduct research and develop innovations that addressed One Health challenges. Innovations were invited from multidisciplinary teams of students who submitted a concept utilizing their collective expertise. Students received support from faculty mentors to develop and execute innovations, which were evaluated collaboratively with study communities to assess feasibility. One example of such an innovation is the improved *tippy tap* with swater reservoir developed by a team of environmental health and engineering students. A *tippy tap* itself is a simple device for hand washing with running water and is very effective in homesteads. The student innovation involved making the tippy tap more efficient and appropriate for high density population areas, such as schools, which necessitated making it more durable with a bigger reservoir for a more sustainable supply of water (Fig. 2). Another group of students developed *The Farmers’ App* (Fig. 3) which is an offline platform that describes clinical signs, symptoms, cause and spread for animal diseases, which has been helpful for farmers in identifying affected animals and seeking veterinary advice. This App was developed collaboratively by students of software engineering, veterinary medicine and environmental health sciences. These and other innovations, including *Bulamu Mobile*, an application that delivers the location of health services (developed by a team led by students of Biomedical Lab Technology), and the *Urban Organic Agriculture* (developed by a team led by a Bachelor of Science in Wildlife Health and Management), were frequently presented at conferences and exhibitions for wider distribution. Research awards also supported dissertation work for many students, examples of which are provided in Table 3.

**Table 3:** Examples of student dissertations supported by One Health research awards

No.	College, Program	Title of Dissertation
1	CHS, School of Public Health (SPH) – Master’s in Public Health (MPH)	Factors associated with diarrhea in children under-five after the occurrence of flash-floods and adaptation strategies developed by households in Bwaise, Kampala
2	CHS, SPH – MPH	Evaluation of community-based groups’ capacity to implement disaster risk reduction interventions in the Mt. Elgon Region
3	CHS, Department of Pharmacy—MSc. in Pharmacology	The potential role of rodents in the transmission of leptospira spp. to humans around Queen Elizabeth National Park
4	COVAB, School of Veterinary Medicine and Animal Resources (SVAR) – Master of Science in Livestock Development Planning and Management (MLD)	Investigation of the antimicrobial resistance crossover along the domestic-wildlife interface in scoring calves of cattle and zebras in pastoral communities around Lake Mburo National Park
5	COVAB, SVAR— MLD	Predictive patterns of fascioliasis outbreaks in cattle grazing around the wetlands of Soroti in eastern Uganda
6	College of Business and Managerial Sciences School of Economics (COBAMS) – Master’s in Arts in Economic Policy and Planning	Health implications on children of poor food safety measures amongst slum dwellers in Kampala
7	College of Agriculture and Environment Sciences (CAES) – M.Sc. Animal Science	Safety of insects and insect meal as protein source for poultry and fish feed
8	CHS –M.Sc. Immunology and Clinical Microbiology	Prevalence of *Mycobacterium bovis* and *Mycobacterium tuberculosis* in samples collected at Kisenyi, Kampala

#### Collaborative teaching and cross-college integration of one health in curricula

To further enhance efforts of multidisciplinary collaboration within the University, structures were created to enable faculty to facilitate courses in their areas of expertise in other colleges. For example, students of Bachelors of Environmental Health Sciences at the School of Public Health at CHS are taught food inspection, including animal anatomy and zoonotic diseases by lecturers from COVAB. The OHCEA Secretariat also worked hand-in-hand with university departments especially in CHS and COVAB to ensure that curricula for newly developed programmes (such as International Infectious Disease Management degree and MPH/MBA, a graduate programme focusing on both Public Health and Business and Management) incorporate concepts and principles of One Health. The MPH/MBA is a dual degree programme that aims to bridge the gap between public health and business, through strengthening and expanding leadership to advance a culture of health in business. The graduates will be equipped with business skills to establish sustainable, well-managed institutions to address health threats across the public and private sectors. Through improved curricula, graduates and professionals from such programmes are most likely to adopt One Health approaches in their future work in Uganda and other countries.

### Early evaluation of the one health activities

Evaluation of the activities was done qualitatively based on students’ experiences, focused on the domains and core competencies of One Health for the African region as defined by Amuguni et al. [22]. The nine core competencies evaluated were management; communication; gender, culture and belief; leadership; collaboration and partnership; values and ethics; systems thinking; policy and advocacy; and research. These domains and core competencies were cross-cutting and integrated into all the One Health activities described. The evaluation of the activities indicated that students gained One Health competencies and appreciated the value of interdisciplinary approach to health challenges. For example, students demonstrated the development of systems thinking by appreciating interconnectedness of health and its determinants.

*“As we communicated with people from different disciplines, I got to know how health challenges are interlinked. I acquired more knowledge about diseases, how the environment influences outbreaks, and I am glad I can use this knowledge even outside the field. As a mass communication student, I get the privilege to inform society in order to avoid future community outbreaks.”* Mass Communication student - One Health attachment

Furthermore, students learned the value of collaboration and partnership across disciplines, improvement of communication skills and research values and ethics.

*“Coming from the health sciences / medical field, I thought I knew it all but under the One Health Institute, I have been challenged in that they bring in students we used to look at as distant disciplines but now One Health has brought us together. It has put us at another level of thinking. I have learnt interpersonal and communication skills. In medicine, I used to think that work is all about receiving patients in a facility but now I know the benefits of working close to the community.”* Nursing student - Students One Health Innovation Club

*“I participated in formulating health messages for the prevention of Anthrax. I have appreciated risk communication – I can now break good news and bad news professionally to a community. I also appreciated the necessity to obtain ethical clearance or consent from the community before engaging them.”* Participant, Graduate fellowships

Students’ activities yielded benefits to communities in several ways. Through interviews with communities and their leaders, it was acknowledged that students contributed to solving community challenges as exemplified by narratives below.

“*… classroom students visited and when they reached that classroom it was full of bats … They told us the bats do carry germs that cause diseases like Marburg. The students using local herbs, came up with a concoction that was able to repel bats when it was smeared in the ceiling of the houses rather than using chemicals (which are not eco-friendly) to kill them*.” Field supervisor, One Health attachment

“*When we had the recent cholera outbreak here, we had to awaken people basing on the concept we had received through working with One Health Students. As such, in the fight of cholera, everyone was brought on board; the local councils were on board, the business community were called onboard as the outbreak would affect their work. An outbreak was going to affect the economic activity, so we had to sit with the business people and agreed on how they were going to contribute. We saw things working out better, unlike the previous days when it was an issue for the health workers alone*.” District Health Officer

Extensive evaluation of the initiatives and related activities, including impacts, are in progress to provide in-depth understanding of their long-term benefits at Makerere University and beyond.

### Achievements, challenges and opportunities

Initial achievements have demonstrated that the efforts Makerere University has made are promoting One Health. For example, all of the colleges in the University actively participated in at least one of the activities implemented by OHCEA, and each had a representative on the executive committee of the Students’ One Health Innovation Club. Students and facilitators involved in One Health activities were also selected University-wide.

To sustain capacity strengthening efforts and engage government in pre-service One Health capacity building, OHCEA and Makerere University had to identify appropriate environments in which trainees could study One Health, and subsequently needed to support the creation of field sites in different districts in Uganda. The One Health field sites were selected primarily to illustrate intense interactions among humans, domestic animals and wildlife, as well as with the environment. Such sites were potential ‘hot spots’ for zoonotic diseases and enabled University students from various disciplines including human and animal health to obtain experiential learning together [23]. In addition, the sites provided an enabling research environment for faculty and students, especially for disease surveillance, prevention and control. Relatedly, government district-level personnel, specifically those from environment, human and animal health offices, were trained and engaged in the supervision of the field attachments. The engagement of district level officers created awareness and recruited them to be advocates for One Health practice. The field attachments offered an opportunity for real-time community-oriented training, which besides building much-needed capacity for the students, was a great way of enhancing relevancy and impact of training institutions for the health of the communities they serve.

One major challenge has been that the application of the One Health approach remains more focused on infectious diseases, especially zoonoses, with a limited consideration of other health challenges. This requires a move to extend the One Health focus to explicitly embrace other health issues including maternal health, water, sanitation, hygiene, environmental degradation, and other issues relevant to global health [15]. Additionally, although One Health has taken some root in different colleges at Makerere University, the most noticeable engagements have been in COVAB and CHS (especially the School of Public Health and the Department of Nursing). Relatedly, there has been some difficulty in incorporating One Health into existing curricula of some programmes from other departments in the University, one of the reasons being the perception that One Health is about only animal and human diseases.

Other challenges related to the University systems, including different programme structures, which may have a significant impact on how fast a new component can be adopted into the University training. For example, curricular review for many undergraduate programmes only occur every 5 years, which could cause delays in making timely adjustments or adding important offerings in One Health. In addition, concerns have been raised that trainings in one health have only been able to involve a small number of trainees relative to the demand for well-trained individuals that are urgently needed in the workplace. Future efforts for enhancing in-service training may rapidly expand the One Health workforce and yield significant synergies with pre-service initiatives, such as those illustrated here.

Through networks and consortia, such as OHCEA and ‘Afrique One’, collaborative support in innovative teaching and the application of One Health approaches can be exponentially expanded, including providing opportunities for trainees and faculty to build cadres of professionals and collaborators early in their careers for research and capacity building across regions [23, 24]. Importantly, the One Health initiatives and experiences detailed here can be used to guide future institutional processes related to development of similar initiatives at local, regional and global scales. Overall, the initiatives demonstrate significant opportunities for students’ exposure and appreciation of One Health domains and core competencies [22] which are key to collaborative global health problem solving at the different levels.

## Conclusion

Integrating One Health approaches into University teaching is feasible and can set the trainees on a path for life-long multidisciplinary collaboration. Graduates exhibit critical core competencies for strengthening national health systems and developing global health innovations in the private sector. Opportunities and challenges identified in our paper at community, institutional and national levels should be taken into consideration when shaping future training and One Health practice for animal, human, plant and environmental health.

## Data Availability

Not applicable.
